# The libertarian predicament: a plea for action theory

**DOI:** 10.1007/s11229-016-1148-3

**Published:** 2016-07-08

**Authors:** Niels van Miltenburg, Dawa Ometto

**Affiliations:** Department of Philosophy and Religious Sciences, Utrecht University, Janskerkhof 13, 3512 BL Utrecht, The Netherlands

**Keywords:** Free will, Libertarianism, Causal theory of action, Compatibilism, Agent causation

## Abstract

Libertarians in the contemporary free will debate find themselves under attack from two angles. They face the challenge of defending the *necessity* of indeterminism for freedom against the philosophical mainstream position of compatibilism. And second, they are increasingly forced to argue for the very *possibility* of indeterministic free will, in the face of the so-called luck objection. Many contemporary libertarians try to overcome the second problem by adopting the causal theory of action (CTA). We argue that this move at the same time undermines their ability to answer the first challenge. On the basis of this, we suggest that CTA might be a theory of action that is biased towards compatibilism. We thus argue that the best strategy for the libertarian is to insist that intentional action itself requires indeterminism. Recent agent-causal accounts offer a promising way of developing such an alternative libertarianism, but we argue that they currently suffer from problems similar to the ones we identify for the event-causalist. If we are correct, then this has an important implication for the contemporary free will debate: action theory should (once again) take centre stage.

## Introduction

In the current free will debate libertarianism, the view that we have free will and that this requires indeterminism, is a problem-wrought position.^1^ Libertarians come under attack from two angles. First, they face the challenge of defending the *necessity* of indeterminism for freedom against the philosophical mainstream position of compatibilism. And second, they are increasingly forced to argue for the very *possibility* of indeterministic free will against powerful varieties of the so-called luck objection. It is therefore fair to say that the contemporary libertarian finds herself on the defensive, or even that she is fighting a rear-guard battle. In this paper, we want to offer a diagnosis of why the libertarian is in this predicament, and suggest an alternative strategy that may restore libertarianism to the vanguard of the debate.

In Sect. 2, we give an overview of how libertarians currently try to answer the two challenges we identified above. As we will see, the standard way to argue for the necessity of indeterminism is by an appeal to van Inwagen’s (1986) famous consequence argument. Libertarians have devised various responses to the second challenge, that of defending the possibility of indeterministic free will against the luck objection. We focus on what many regard as the most promising response that has recently been clearly formulated by Franklin (2011a). This response insists that luck cannot diminish an agent’s control over her action because the kind of control at issue is what we call *agential* or *intentional* control. And, Franklin argues, the libertarian can account for that kind of control in terms of causation by mental states—that is, by adopting the causal theory of action (CTA).

We believe that there is something about this response to the luck objection that is essentially correct: it *is* enough to point out that an agent has intentional control over an action in order to rule out luck in a freedom-destroying sense. However, we argue that it is problematic for the libertarian to spell out such intentional control in terms of CTA because there is a strong tension between rejecting the luck objection on the basis of CTA on the one hand, and rejecting compatibilism on the basis of the consequence argument on the other. Now instead of arguing that determinism diminishes agential control, the libertarian might argue that indeterminism is necessary to *enhance* that control sufficiently for an action to be free. In Sect. 4, we argue that the enhancement-strategy is severely limited.

Thus it seems that the libertarian has painted herself into a corner. By adopting CTA in response to the second challenge, she undermines her ability to answer the first challenge. This may seem like good news to the compatibilist, and indeed, it might be one explanation of the relative unpopularity of libertarianism. However, we believe that there is more to be said on behalf of the libertarian. In Sect. 5, we focus on a pervasive but little noticed methodological assumption underlying the free will debate: that the question in the debate should be what, if anything, must be added to a mere *intentional* action in order to render it a *free* action. We believe the contemporary libertarian’s problems stem from conformation to this way of conducting the debate. Thus we suggest

that the libertarian would be better suited to follow an alternative and more radical strategy: to reject CTA, and argue that intentional action itself requires indeterminism. If this is right, it follows that event-causal libertarianism is ultimately an unstable position.

Given our critical stance on event-causal libertarianism, one might expect that we have a close ally in agent-causal libertarian accounts. However, we will argue that most extant agent-causal theories fall into the same trap as the event-causalist: agent causation is seen as an extra ingredient needed in addition to intentionality. We then explore the desiderata of an agent-causal theory that might avoid this problem. Developing such a theory will require more fundamental work in the philosophy of action. But we can already see that the resultant variety of libertarianism will be, in a sense we explain, more radical than either event-causal libertarianism or most varieties of agent causation.

## Libertarianism and the free will problem

Philosophical thinking about free will often starts with reflections about determinism. How can our actions be up to us if it was necessary that we would perform them even before we have decided what to do? Indeed, as van Inwagen (1986) argues, freedom and determinism (or liberty and necessity as they were historically called) were once seen as strict opposites. Thus traditionally the free will problem was to reconcile human freedom with the deterministic workings of nature. The main division in the debate is therefore between compatibilists, who believe this reconciliation can be achieved, and incompatibilists, who deny this. These days there exist many sophisticated varieties of compatibilism, and the incompatibilist therefore has serious work to do in explaining why she believes free will requires indeterminism. The most important incompatibilist argument in the contemporary debate has been van Inwagen’s consequence argument, which, in its informal formulation,^2^ runs as follows:

If determinism is true, then our acts are the consequences of the laws of nature and events in the remote past. But it is not up to us what went on before we were born, and neither is it up to us what the laws of nature are. Therefore, the consequences of these things (including our present acts) are not up to us. (van Inwagen 1986, p. 16)

What this argument brings out is that a deterministic world view makes it hard to comprehend how we can still be the authors of our own actions, or how our actions can be “up to us”. However, the sense in which free will requires that we control our actions is, not surprisingly, itself a point of philosophical contention. Hence, compatibilists can overcome the consequence argument if they show that there is a relevant sense in which an agent’s acts can be up to her even when the occurrence of these acts is already determined. We return to the consequence argument, and the question what is the right sense of ‘control’, in Sect. 5. For now, it is enough to note that van Inwagen’s argument remains strongly contended.

However, in recent years it has become increasingly clear that the problem of free will is not exhausted by worries about determinism. Those incompatibilists who are libertarians, believing that human actions are undetermined and free, also face a difficult challenge. For finding out that human actions are not determined would not, by itself, help much in understanding how our actions can be free. In fact, indeterminism might make it even harder to understand how we can exert control over our own actions. Indeterminism, some would argue, merely injects randomness into the world. Hence, if human action is indeterministic, what we do seems to be a mere matter of chance, similar to the outcome of a dice roll or a coin flip. And if there is anything we cannot control, it is the outcome of a chancy process. To illustrate this “problem about luck for libertarians”, or the luck objection as we will call it, Mele (2006) asks us to imagine two worlds, $W_{1}$ and $W_{2}$, that have the exact same laws of nature and up to now have had the exact same past. Now consider Joe, a free agent that decides to *A* in world $W_{1}$. According to the libertarian, Joe can decide not to *A* in world $W_{2}$. The libertarian, after all, does not believe that the past and the laws of nature fix current decisions. But, Mele complains:

If there is nothing about Joe’s powers, capacities, states of mind, moral character, and the like in either world that accounts for this difference, then the difference seems to be just a matter of luck. (Mele 2006, p. 9)

If our choices are just matters of luck, then what we decide is up to chance, and hence cannot be up to us. Thus an indeterministic world view also makes it hard to understand how we can be the authors of our own actions, how we can control what we do. Hence the libertarian is faced with not one, but two challenges. A good libertarian theory of free will has to (1) explain why indeterminism is *necessary* for free action, and (2) explain how free action is *possible* under indeterminism.

## Luck, control, and CTA

Let us start by considering the way the libertarian, in the contemporary free will debate, has attempted to overcome the luck objection. At first sight, it might seem that the luck problem is not too hard to escape. Consider Mele’s free agent Joe. In one world Joe decides to *A*, and in the other he decides not to *A*. Now even though there might be nothing “about Joe’s powers, capacities, states of mind, moral character, and the like” that explains this difference, we do have one explanation for why Joe *A*’d in the one world and did not do so in the other. That is, he *decided* to *A* in the one world and decided not to *A* in the other. Mele is right that nothing can explain the occurrence or non-occurrence of *A* other than Joe’s decision. But this is no argument against the libertarian, since it is exactly her position that we have this undetermined capacity to shape the future according to our own decisions.^3^ Even if this is an adequate reply to Mele, it unfortunately does not fully repel the luck problem. To bring this out we should look at perhaps the most poignant explication of the worry that indeterminism precludes freedom: van Inwagen’s (2000) roll back argument.

Van Inwagen considers Alice, a free agent who is torn between telling the truth and lying, but in the end tells the truth. If libertarians are right, Alice could also have lied. Therefore, if God were to roll back the universe to the point right before Alice decided to tell the truth, and let things proceed again, she might end up lying. Imagine that God does this a large number of times, say a thousand. Then we would get a certain ratio of, e.g., three hundred lies to seven hundred truth-tellings. But this makes it seem that there just is a 70 % probability that Alice tells the truth and a 30 % chance that she lies. However, if Alice’s actions are just a matter of chance, then it does not seem to be something over which she has any control, i.e., her original telling of the truth was no free action after all.^4^

What the roll back argument brings out is that the reason why indeterminism threatens freedom is not just that an action might lack an explanation (as Mele’s put it), but that an agent would lack control over what she decides to do, just as she would lack control over the outcome of a die roll. The problem van Inwagen sketches directly threatens Alice’s ability to control what she does by arguing that what she does is a mere matter of objective probability.^5^

Franklin (2011a) has recently argued that the libertarian can escape the problem by endorsing the causal theory of action (CTA). He analyses the roll back argument as consisting of two basic steps:

1. If human actions are undetermined, then they are a matter of chance.
2. If actions are matter of chance then they are not free.

According to Franklin (1) is obviously correct: “All undetermined actions have an objective probability of less than 1 of occurring and so all their occurrences are a matter of chance” (Franklin 2011a, p. 216).^6^ But he disagrees with (2): the fact that human actions are a matter of chance does not imply that we have no control over them, and hence that we are unfree.

This is where CTA comes into play. According to CTA, intentional actions are distinguished from mere bodily movements (like the knee reflex) by their causal history.^7^ Intentional actions are events that are caused, in the right way, by agent-involving mental events—this is why the theory is also referred to as ‘event-causalism’.^8^ Although proponents of CTA differ in what kind of mental events are supposed to do the causing (beliefs, desires, commitments, intentions) they generally agree that these mental events are identical to, or that their contents represent, the agent’s reasons.^9^ For our (and Franklin’s) purposes what is important is that on this account of action an agent’s exercise of agential control over her actions consists in her actions being appropriately caused by her mental states. An agent does not control the movement of her lower leg if it is caused by a doctor who strikes her patellar ligament with a reflex hammer, but she does control the movement if it is caused by, say, her desire to kick the doctor.

Event-causal *libertarians* add to this story by claiming that the causation involved in the production of action is non-deterministic causation. Alice, for instance, can both tell the truth or lie, because both her reasons for truth telling as well as her reasons for lying could become causally active. Whether Alice tells the truth or lies is, however, not totally random. It rather is the case that her reasons for each action come with a certain strength corresponding to their probability of causing the action. Hence, if Alice has stronger reasons to tell the truth than to lie, this translates into a higher probability that she will tell the truth compared to her probability of lying. On the event-causal libertarian’s view then, deciding just is a matter of non-deterministic causation. The decision to tell the truth *consists* in the fact that Alice’s reasons for truth telling cause her action.^10^ But Alice could also have decided to lie, since there was an objective probability that Alice’s reasons for lying had become causally active instead of her reasons for telling the truth. Now it is important to note that whatever Alice ends up doing, it will always be *her* reasons—either those for, or those against telling the truth—that do the causing. Furthermore, since CTA tells us that the exercise of agential control just consist in an agent’s reasons causing her actions, Alice is in control whatever she ends up doing. Hence, what she does *is* up to her, and the roll-back argument loses its bite.

To some this reply may seem unsatisfactory. Schlosser (2014), for instance, argues that Franklin merely has shown that luck neither threatens Alice’s ability to tell the truth nor her ability to lie, but that it remains a matter of luck *which* ability she will exercise. Alice, he states, “lacks the power or control to exercise either one of the two abilities such that she can select which alternative to pursue” (Schlosser 2014, p. 380). We believe that this complaint betrays a failure to comprehend the role CTA plays in Franklin’s event-causal libertarianism. CTA provides an *account* of what it *is* for an agent to select one alternative over another. On CTA, there is nothing more to selecting an alternative than just performing one action instead of another. Agents thus control which ability they exercise by simply exercising that ability on the basis of their reasons for exercising it. So we are willing to accept that Franklin has successfully shown that if CTA is the correct story about agential control, then there is no ground for the claim that an agent’s action is outside of her control, even if the causation involved is probabilistic rather than deterministic. However, whether Franklin’s argument is ultimately a successful reply to the luck objection or not is not at issue here. For we want to argue that *if*, or to the extent that, Franklin is correct, this reply to the challenge of explaining the *possibility* of indeterministic freedom makes it very difficult for the libertarian to respond to the first challenge we identified: that of arguing for the *necessity* of indeterminism to freedom.

Why does Franklin’s appeal to CTA in order to solve the luck objection backfire? As we have seen, what does the work in the luck objection is the idea that chance threatens agential control. The solution is to *account* for agential control in a way that shows this idea to be misleading—namely, in terms of causation by an agent’s reasons. However, the consequence argument—which arguably is the main argument most libertarians offer to establish the necessity of indeterminism—also relies on the idea that agential control is undermined, but this time by determinism instead of by chance. What, then, is there to prevent the compatibilist from arguing against the consequence argument, analogously to Franklin, that it is mistaken to think that determinism undermines agential control, because such control *just is* an action’s being caused by reason states? For if CTA correctly construes agential control in terms of the agent’s reasons causing her action, then this does not seem to depend on whether the causation is deterministic or not.^11^ Consider Charlie, who inhabits a deterministic world. It has always been determined, even before Charlie was born, that today at six, she will go for a drink. The consequence argument questions whether Charlie’s going for a drink is really up to her if it was already fixed. But with CTA in hand the compatibilist can argue that the answer to this question depends on the causal route that leads up to Charlie’s action. If Charlie is remotely controlled by an evil neuroscientist who steers her to the bar, then her going for a drink is not up to her. But if, on the other hand, her going to the bar is caused by, say, her own desire for refreshment, then Charlie did control her action. After all, Charlie’s exercise of agential control just *consists* in her reasons causing her action.

The point is even clearer if we remind ourselves that one of van Inwagen’s more formal renderings of the consequence argument depends crucially on the modal operator $\mathrm {N}$, which he asks us to read as ‘no one has or had a choice about ____’. The argument then proceeds as follows. Since no one ever had a choice about the laws of nature and the initial conditions of the universe, and under determinism, the laws and past fix any action *p*, it follows that $\mathrm {N}p$. To establish this conclusion, van Inwagen employs two famous inference rules. The first is $\alpha$, which states that $\Box p \vdash \mathrm {N}p$. The second is the (in)famous rule $\beta$, which states that $\mathrm {N}(p \rightarrow q), \mathrm {N}p \vdash \mathrm {N}q$. It seems safe to say that most of the debate concerning the consequence argument has focused on the merits of $\beta$.^12^ However, we can now see that arguing against $\beta$ is not necessary to challenge van Inwagen’s argument. For we should note that CTA is precisely an account of the very notion with which $\mathrm {N}$ is concerned—the notion of *choice*, or of someone’s doing something *by* choice.^13^ And on the account that CTA gives of this notion, it is simply not true that if it is necessary (determined) that *p*, no one had a choice about *p*. For *p* may be an event caused by an agent’s reasons, and if so, it would count as the agent’s choice in virtue of that. So it seems that CTA and the *prima facie* plausible rule $\alpha$ are at odds. This, it seems, is what John Bishop had in mind when he argued:

The Consequence Argument is false because the core claim of the Causal Theory of Action is true. [...] if the agent’s behavior, which is the *deductive* consequence of these unavoidable states of affairs, is also the *causal* consequence of the right kind of states of the agent, the agent’s behavior will fulfil conditions sufficient for it to count as action. If facts about the remote past cause present behavior via the right sort of causal chain, the causal consequence of what is unavoidable will actually constitute a case of something that comes about through agent-control. (Bishop 1989, p. 57)^14^

If our argument that CTA deals with both the luck problem and the consequence argument in one fell swoop holds, then it seems fair to raise the question why many philosophers in the free will debate keep insisting on the incompatibility of free will and (in)determinism. As soon as the libertarian accepts CTA, she is left wondering why she thought that freedom required indeterminism in the first place. In other words, if the libertarian explains the *possibility* of free action under indeterminism with the help of CTA, she is hard pressed to defend the *necessity* of indeterminism for free action. CTA simply explains how agents can control their actions in both deterministic and indeterministic universes. If that is right, it seems that event-causal libertarianism is a paradoxical position.^15^ But might the event-causal libertarian not justify the indeterminism requirement in another way? In the next section we will explore that suggestion.

## The problem of enhanced control

We have suggested that the libertarian has painted herself into a corner by her reply to the luck objection. For that reply offers the compatibilist an easy way out of the consequence argument. This makes it impossible for the libertarian to argue for the necessity of indeterminism on the basis of that argument. However, Franklin (2011b) is hopeful that an argument can be found that shows that the agential control already available to the compatibilist would be meaningfully improved by indeterminism.

On Franklin’s view, the challenge is to show that freedom requires such enhanced control. It is important to see how this contrasts with a suggestion by Mele (2006), who has argued that the libertarian might even grant that freedom does not require more control than is available under determinism. Still, Mele proposes, indeterminism might play a meaningfully enhancing role. According to the position he calls “soft libertarianism”, the majority of our actions might be deterministically caused, and nevertheless free. But, he claims, we might still take our actions to have a more *valuable* freedom if they were undetermined:

Unlike hard libertarians, soft libertarians leave it open that determinism is compatible with our actions’ being up to us in a way conducive to freedom and moral responsibility. However, they believe that a more desirable freedom and moral responsibility require that our actions not be parts of the unfolding of deterministic chains of events that were in progress even before we were born. If soft libertarians can view themselves as making some choices or decisions that are not deterministically caused [...] then they can view themselves as initiating some causal processes that are not intermediate links in a long deterministic causal chain extending back near the big bang. (Mele 2006, p. 97)

There are two things to observe about Mele’s strategy for defending the requirement of indeterminism. First, if soft libertarianism possibly allows the compatibility of freedom and determinism, it is questionable to what extent soft libertarianism is a form of libertarianism at all.

Second, even if we grant that it may be a position related to libertarianism, we should note how weak the soft libertarian’s reason for preferring indeterminism is. The argument Mele offers for valuing undetermined actions over determined ones seems to run in a very small circle. He says that it is desirable for an agent that her action is undetermined because she can then view it as a “causal process that is not an intermediate link in a deterministic causal chain”. But that is just to say that she can view her action as something that is, precisely, undetermined. Mele’s suggestion, then, seems to come down to the thought that indeterminism is valuable for indeterminism’s sake.

Now Franklin believes he can give a superior solution to the problem of enhanced control, that firmly sticks to libertarianism. To do so, he proposes an ‘argument from opportunity’:

It is often mistakenly assumed that an agent’s control is wholly exhausted by the agent’s powers and abilities. I argue, however, that control is constituted not just by what we have the ability to do, but also by what we have the opportunity to do. (Franklin 2011b, p. 687)

Franklin makes it very clear that he intends the notion of an opportunity to be very robust. That is, what it is for agent to have the opportunity to do *X* is not, merely that she will do or would have done *X* if she decides to do it or had so decided. Neither is it the case that an agent has the opportunity to do *X* if she only believes or thinks that she can do *X*, i.e. an opportunity is not a doxastic possibility. Rather, for an agent to have the opportunity to do *X* means that it is really possible for her to do it. Now Franklin argues at length that deterministic agents never have the opportunity to do otherwise. In an indeterministic universe, agents would thus have more opportunities, and hence more control.

However, the thesis that deterministic agents never have the opportunity to do otherwise seems to be true just in virtue of the way that the notion of an opportunity has been defined: if an opportunity to do *X* is the ‘real possibility’ of doing *X*, it is just the very definition of determinism that an agent never has more than one opportunity. That, of course, does not yet give us any reason for thinking that having multiple opportunities—i.e., indeterminism—is relevant to control. Given Franklin’s criticism of Mele, it is surprising to see how little he can offer in defence of that idea:

The opportunity to do otherwise is not simply another opportunity on top of the many opportunities that compatibilist agents already possess. Rather, it is a significant addition. It affords agents with the opportunity to direct their lives in more than one way, to author how their lives unfold, and to choose from among several causally open options, thereby taking a stand on the kind of person they will become. This is no trivial addition. Indeterminism, therefore, is relevant to enhancing control because its existence is necessary for agents to possess the freedom to do otherwise. (Franklin 2011b, p. 704)

This seems to say that indeterminism is a necessary requirement of free will because its existence is necessary for agents to possess the opportunity to do otherwise. But the opportunity to do otherwise just *is* the real possibility to do otherwise, and that just *is* the presence of indeterminism. So we are again tracing a small circle.

Franklin then calls having multiple opportunities a “significant addition”, because without it agents could not “choose from among several causally open options”. But again, that is obviously circular: for several options for action to be causally open just is for them to be physically undetermined, and hence cannot be a reason for insisting on indeterminism. And the rhetorical evocation of the significance of “taking a stand on the kind of person [an agent] will become” is hollow—the compatibilist will agree, but simply give a deterministic interpretation.^16^

Also notice that, even if he was able to show that having multiple opportunities is really control-enhancing, Franklin would still need an argument to show that only agents who possess such enhanced control are free. Otherwise, his position would collapse into Mele’s soft libertarianism. But Franklin offers no argument to think that enhanced control, as he construes it, would be a necessary requirement of free will, above the agential control already provided by CTA. Thus the argument for the *necessity* of indeterminism on the ground that it is freedom enhancing remains unconvincing.

## Intentional action and freedom

In the previous section we have argued that both Franklin and Mele do not give a satisfactory answer to the problem of enhanced control. This, of course, does not establish that no such answer can be given whatsoever, but we believe the difficult situation the libertarian finds herself in should give us pause. Although the compatibilist will see this difficulty as good news, we believe that there is more to be said on behalf of the libertarian. In particular, we suggest we need to reflect on the specific assumption about the role that indeterminism plays in contemporary libertarian accounts. We argue that this will help to uncover a more fruitful strategy for the libertarian.

Indeterminism, for the event-causal libertarian, plays the role of an *extra ingredient* needed for “transforming a mere action into a free action” (Franklin 2011a, p. 203).^17^ On this picture, free actions are a subset of the larger class of (intentional) actions—a subset that fulfils some extra conditions. And the class of intentional actions is, presumably, marked off from the yet larger class of bodily movements by other criteria.^18^ Philosophers who talk about such a transformative ingredient thus believe that the difference between a free and an unfree action only depends on factors *extrinsic* to the action’s intentionality. Traditional compatibilists, for instance, argue that an agent’s intentional action is free if she is not coerced to perform it.

But it is important to note that this way of construing the free will debate—as a search for the requisite extra features that turn mere intentional action into free action—is optional. The easiest way to see this is by comparing it with a different kind of compatibilist account, which takes freedom to be an *intrinsic* feature of intentional action. The thought that free will does not require anything in addition to intentionality—that it is inherent to intentionality itself—has been defended by Donald Davidson, the contemporary progenitor of CTA. In his ‘Freedom to Act’, Davidson tries to analyse ‘*A* is free to do *x*’ as ‘he would do *x* intentionally if he had attitudes that rationalised his doing *x*’ (Davidson 1973, p. 148). In other words, the freedom to do something just lies in the ability—or as he calls it, “causal power”—to do it intentionally. Although Davidson ultimately rejects this specific analysis because the problem of deviant causation makes it impossible to empirically identify the causal conditions of intentional action, he believes that this is “no obstacle to the view that freedom to act is a causal power of the agent” (Davidson 1973, p. 155). It seems, then, that Davidson believed that there is no more to free will than the ability to act on the (causal) basis of one’s own reasons. And on his account, being caused to act by one’s own reasons is what intentional action consists in.

If we assume that Davidson was right that intentional action is intrinsically free, this opens up an intriguing new perspective on the free will debate, and in particular on the predicament of the libertarian. For if CTA, as an account of agential control, just is an account of freedom, then it would seem that adopting CTA already is to answer the question whether freedom and determinism are compatible in the affirmative (as Davidson indeed insists). That would explain why the event-causal libertarian’s appeal to CTA as a response to the luck objection backfires by making it impossible to argue for the necessity of indeterminism: she is looking for reasons to demand indeterminism after having already bought into a compatibilist theory of action.^19^ Or to put it differently, the problem of the libertarian in the current debate is that she construes free action as follows:

**An action is free iff:**

1. The action was performed for a reason, and
2. The action was not predetermined.

However, on the account of acting for a reason the contemporary libertarian accepts condition (2) is logically independent of condition (1): there is nothing in the notion of causation by reason-states which excludes determinism.

If this diagnosis is correct, it suggests a more fruitful strategy for the libertarian. First, she should reject the idea that freedom consists in extrinsic features of intentional action, and agree with Davidson that intentional, agential control just is freedom. Second, she should reject CTA as an inherently compatibilist theory of such agential control. Finally, she should offer an alternative theory of action, which shows intentional action to be incompatible with determinism. She should not just be an incompatibilist about free will and determinism, but rather about agency and determinism. That is, the libertarian should not believe that determinism *diminishes* control (or that indeterminism enhances it), but that determinism *destroys* control.

## Libertarianism and agent causation

We have so far argued that the game for the libertarian becomes very difficult as soon as she accepts the intrinsically compatibilist causal theory of action. We therefore believe that libertarian thus should seek refuge with an altogether different account of action. In recent years just such an alternative to event-causalism has been (re-)gaining popularity. We are, of course, talking about agent-causalism. Unfortunately, we will argue, the main agent-causal theories in the current free will debate fall into the same trap as their event-causal counterparts: they propose agent causation as a mere extra factor that turns intentional action into free action. Therefore they, too, are unable to argue for the necessity of indeterminism. Nevertheless, we believe that if this pitfall is avoided, agent-causalism might in principle provide the agency incompatibilism the radical libertarian aims for.

Agent-causalism is the view that free actions, or the volitions leading up to them, are directly brought about by the agent. Such agent causation is radically different from and cannot be reduced to event-causation. The reason why agent-causalism is especially appealing to the libertarian is that it, *prima facie*, nicely captures and explains how human actions are not predetermined. An agent, it is argued, is not the kind of thing that can itself be caused (although events involving the agent, like her birth, can be). Therefore when an agent causes her action or volition, she is “in a strict and literal sense” (Clarke 2003, p. 134) the ultimate source or un-caused cause of the event. In this way agent-caused actions are truly up to the agent and therefore free.

Of course the major challenge for the agent-causalist is to explain this irreducible notion of agent causation. Many philosophers think that the concept of agent causation is utterly impenetrable. Appealing to that concept to clear up the mystery of free will seems nothing more than “giving a name to a mystery” (Nagel 1986, p. 115).^20^ Adding to the obscurity is the fact that agent-causalists traditionally have alluded to the nature of agent causation by contrasting it with the causality exhibited in the inanimate world.^21^ For this reason agent causalism has long been seen as an untenable position. In recent years however, some have argued that agent causation is not as strangely unique as it might *prima facie* seem. O’Connor (2009) for instance, defends a view of agent causation in terms of powers. An agent exercises her power to act, just like, for instance, water can exercise its power to dissolve salt. The agent causation involved in free acting, he argues, is no more mysterious than the causation involved in the manifestations of the powers of inanimate objects. Clarke (2003, pp. 185–218) and Lowe (2008, pp. 143–147) similarly argue that agent causation (or rather substance-causation, as it is called when we consider the powers of non-rational or inanimate objects) might actually be a ubiquitous phenomenon.

We will not here discuss whether this strategy of demystifying the concept of agent causation is successful or not. What we want to point out is a tension between this idea of substance causation as a pervasive natural phenomenon and the idea that it provides agents with freedom. For if inanimate, impersonal entities can also exercise substance-causation, then they too seem to be the strict and literal sources of their effects. But no one, of course, wants to claim that, say, the dissociation of salt is something that is up to the water in the sense that is relevant to free will. Hence it seems that the fact that an action (or any other event) is substance-caused is logically independent from it being under the agent’s agential control.

And indeed, the main contemporary agent-causalists explicitely think of agent causation in separation from intentionality. O’Connor, for instance, writes: “Agent causation is a necessary feature of freely chosen activity, even though there may be possible forms of intentional activity that lack it altogether” (O’Connor 2011, p. 311).^22^ Clarke (2003, pp. 133–150) even argues that we must supplement the agent-causal account of the causation of action with the standard event-causal account of acting for reasons, i.e. CTA. If our arguments so far have been correct, then accepting CTA should be a non-starter for libertarians because it is an inherently compatibilist theory.

Thus the main contemporary versions of agent-causalism do not provide us with the alternative account of action that we believe the libertarian requires. It is thus better to understand agent causation as an answer to the problem of enhanced control. This is sometimes explicitly the purpose of agent-causal theories, for instance in Clarke (2003, p. 93).^23^ But it seems that agent causation is not really that enhancing, for, as we have seen, it also is exhibited in inanimate processes.

A further problem for the agent-causal libertarian is that there is no good reason to suppose that substance causation cannot be deterministic. The dissociation of salt, for instance seems to be a deterministic process. And indeed the idea of deterministic agent causation has been recently defended by compatibilists.^24^ Hence, even if agent causation is necessary for free will, the agent-causal libertarian still needs a separate argument for the necessity of indeterminism. We can conclude that the contemporary agent-causal libertarian is in a predicament similar to that of her event-causal counterpart. To argue for the *possibility* of agent-causation she defends that it is a phenomenon present everywhere, even in deterministic parts of nature. But this potentially undermines her argument for the claim that the agent-causation of free action has to be *necessarily* indeterministic.^25^

We believe that the root of the problem is that the contemporary agent-causal libertarian, just like the event-causalist, construes free action decompositionally. But instead of two logically independent requirements the agent-causalist has three:

**An action is free iff:**

1. The action was performed for a reason,
2. The action was not predetermined, and
3. The action was agent-caused.

It seems to us that the agent-causal libertarian has trouble motivating the need for any of these requirements precisely because she views them as independent. But we believe that there is no principled reason to do so. As a matter of fact, Helen Steward has recently defended an agent causal theory that unifies requirement 2 and 3. Her theory starts from the idea that agents (including animal agents) can cause things by moving themselves. According to Steward, when an agent moves herself she is by definition not moved by something else, and therefore her self-movements are up to her. Thus the very idea that a movement can be up to an animal is incompatible with determinism; for to say that something is up to an animal is to say that the animal can *settle* that matter. And it is impossible to settle something that has already been settled before. Therefore, Steward argues, if there is to be agency at all, the past and laws of nature cannot already fix what the agent does. Hence demand (2) and (3), are not independent: the requirement that an action is agent-caused contains the requirement that it is not pre-determined. Steward thus defends a form of ‘agency incompatibilism’, the position that we, at the end of the last section, proposed is the most promising for libertarians. However, we believe that Steward’s account ultimately runs into trouble because she does not explain how indeterministic agent causation and acting for a reason hang together. In other words, it fails because she does not engage with action theory.

First of all, even if agent causation essentially involves settling the undetermined, then this would still not be sufficient for free will. For if it were, then a radium atom that by decaying settles the clicking of a Geiger counter would also be free. Perhaps Steward can object to this that the radium atom does not really agent-cause the decay in the same way as animals cause their self-movements, if decay is an agent/substance causal process at all. And indeed Steward admits that she needs to say more about the nature of settling in order to “sustain the idea that an animal may be truly in charge of what it does” (Steward 2012, p. 198). She does so by fleshing out her particular views on agent causation. Agent causation, on her account, is a form of *top-down* control. An agent’s input does not happen prior to the neuronal processes that initiate her movement, nor is it identifiable with these neuronal processes. Rather, the role of the agent is to monitor and control the entire process of action. But in order to render this idea of top-down causation plausible she, similar to O’Connor and Clarke, argues that it is present everywhere in nature:

For example, a cell is a structure that, once formed, can be a source of control over the chemical processes that go on within it in the sense that laws and principles that belong to the level of the cell overtake those that belong to the level of the molecule when it comes to understanding how those lower-level processes are integrated and harmonised to serve the purposes of the cell. (Steward 2012, p. 245)

The problem with this approach is that if being in charge of what you do consists in top-down control, and a cell can also exhibit top-down control, then it again becomes unclear why agent causation requires indeterminism. For it is not clear that the behaviour of an individual cell needs to be indeterministic. And even if one does want to argue that all biological processes involve indeterminism, the problem remains. For Steward believes that top down control is also exhibited in processes that clearly seem deterministic, such as the motions of water molecules in a whirlpool or the persistence of familiar objects. Hence, it seems that, by pointing out that top-down agent causation occurs everywhere in nature, Steward again severs the connection between requirement (2) and (3).^26^

Perhaps the agency incompatibilist can find a way out of this conundrum if she explains how the causation involved in agency is different from the top-down control that is exercised by other entities. The key difference, we believe, is that agents control their movements by acting on the basis of *reasons*. Steward cannot distinguish between free action and ‘mere’ top-down causation in this way, since she denies that only purposive actions can be self-movements or settlings.^27^ That, we think, is a mistake. If agent-causalism holds a promise for the radical libertarian, she is challenged to give an agent-causal account of what it is to act for a reason. It will not do to outsource the question what it is to act for reasons to CTA, as e.g. Clarke does. Nor will it do to leave that question open, as Steward does. Rather, the libertarian will have to explain how requirements (2) and (3) are conceptually connected to (1). Unfortunately, we cannot here develop an account of agency that would satisfy these desiderata. And it remains an open question whether this is possible.^28^ But nevertheless we think that the arguments in this paper have considerable potential to move the debate forward. For, if we are right, the best hope to develop a convincing libertarian theory lies in the pursuit of a theory of action as already involving indeterminism—and not in the quest to show that indeterminism allows for slightly more control than the compatibilist can already offer.

## Concluding remarks

In this paper we started out by sketching the predicament the contemporary libertarian is in: she has to fight a two-front battle, arguing for both the *necessity* of indeterminism to free action and the *possibility* of indeterministic free action. We have argued that if the libertarian accepts CTA to fight on the latter front she weakens her position on the former. The problem for such an event-causal libertarian, we have argued, is that she just views indeterminism as a condition that is logically independent from agential control. It might be thought that those who reject event-causal libertarianism in favour of agent-causalism do a better job. But they too cannot escape the predicament as long as they defend that agent-causalism is a requirement of free will that is independent of indeterminism and agential control.

Nevertheless, we have argued that a stronger libertarian position is possible and worth exploring. The libertarian could argue that agency itself, instead of just free agency, is incompatible with determinism. The focus of this more radical libertarian should be on a discussion of agential control itself, rather than on a discussion of extra factors supposedly required in addition to it. What the libertarian needs is a theory of how action already requires indeterminism. Does it then follow from such radical libertarianism that all intentional actions are free? This may seem implausible to some: there is clearly a sense in which someone who acts at gunpoint is less free then someone who is not coerced. However, the radical libertarian can argue that there also exists a more fundamental kind of freedom that is not gradual. That fundamental freedom she calls “free will”, and it is exhibited in every intentional action: it consists in selecting a metaphysically open course of action on the basis of one’s reasons.

Interestingly, such a radical commitment to a fundamentally indeterministic form of freedom is rarely found among current libertarians. At the end of his *Essay on Free Will* Peter van Inwagen, for instance, reluctantly admits:

It is conceivable that science will one day present us with compelling reasons for believing in determinism. Then, and only then, I think, should we become compatibilists. (van Inwagen 1986, p. 223)

Similarly, when Robert Kane is asked by John Martin Fisher what he would do if he were to ever wake up to a headline reading “Scientist have discovered that determinism is true”, he concedes:

If I do ever read Fischer’s future headline and it is true, I would give up my libertarian view and perhaps go over to one of these other views. (Fischer et al. 2007, p. 181)

But why should the libertarian commitment to indeterminism be so frail? If the libertarian truly believes that we have free will and that free will requires indeterminism, she would be forced to deny that free will exists if science proves determinism. However, for the radical libertarian, this would also mean that science has disproved the existence of action itself. And given the crucial role agency plays in both our self-understanding and our engagement with the world, it seems simply inconceivable to the radical libertarian that scientists will ever find compelling evidence for universal determinism. Determinism might be possible, but not in a world that is inhabited by human beings.

## Footnotes

1 In a large survey among professional philosophers in 2009 only 16.7 % of the respondents indicated that they accepted or leaned towards libertarianism. See Bourget and Chalmers (2014).

2 For a good overview of the formal renderings of the consequence argument and possible issues with them, see Chap. 1 of O’Connor (2000). Also see Sect. 3 of this paper for some short remarks on the formal features.

3 Randolph (2003, pp. 77–82) offers a reply similar to this.

4 Recently, Buchak (2013) has argued against this step in van Inwagen’s argument. She argues, to our mind rightly, that from the fact that after any large number of roll backs, there will always be some number of truth-tellings *x*, and some number of lyings *y*, it does not follow that there was some objective probability that Alice would tell the truth. This application of the law of large numbers is only warranted if we already know that the results in each roll back are due to a stochastic process. We ignore this complication here because the assumption that a free decision or action has some determinate objective probability is not actually contested by most contemporary libertarians. In fact, as we will see, many libertarians are positively committed to this assumption through their adherence to the causal theory of action. So interestingly, it seems that the first step of the roll back argument is a mistaken way to establish a premise that most libertarians in the contemporary debate has already granted.

5 Indeed, this is also one of the worries that motivates Mele’s attempt to formulate the luck objection: As soon as any agent [...] judges it best to *A*, objective probabilities for the various decisions open to the agent are set [...]. Larger probabilities get a correspondingly larger segment of a tiny indeterministic neural roulette wheel in the agent’s head than do smaller probabilities. A tiny neural ball bounces along the wheel; its landing in a particular segment is the agent’s making the corresponding decision. (Mele 2006, p. 8)

6 However, see fn. 4.

7 CTA is, of course, famously argued for by Davidson (1963).

8 Some recent versions of CTA are not committed to the idea that events are the causes of actions, e.g. Stout (2010). We believe that the argument of this paper pertains equally to all variants of CTA. Therefore we will ignore this complication, and focus on the event-causal position, which is also the most prominent in the free will debate.

9 Not all contemporary proponents of CTA agree that an intentional action must be causally related to reasons. It might be sufficient if the action is caused by a mere intention. We will ignore this complication because in the example under consideration Alice *is* considering her reasons and forming an intention on the basis of them.

10 Or as Mele put it, the landing of the ball in a particular segment of the neural roulette wheel “is the agent’s making the corresponding decision” (Mele 2006, p. 8), see fn. 5.

11 Indeed, Davidson himself exploits CTA (together with his doctrine of anomalous monism) to argue for the compatibility of determinism and mental causation. See Davidson (1970).

12 For an overview, see Steward (2012, pp. 26–31).

13 Perhaps this often escapes notice because it seems that the prime *analysandum* of CTA is intentional action. And is there not a difference between acting intentionally and making a choice to act? We think it would be mistaken to separate these two notions so strictly. At least for Davidson (1973), an intention or action is the conclusion of practical reasoning, and so the result of an agent’s considering the question what to do—her making a choice. In fact, some varieties of CTA explicitly postulate *decision* states as intermediaries between an agent’s reasons and her action.

14 Bishop claims no originality for this line of arguing against the consequence argument but attributes it to Slote (1982). However, it is Bishop himself who points out the centrality of CTA for compatibilism. In Sects. 2.4–2.5 of his book he even defends his opinion that “a positive case for the falsity of [the consequence argument] can be achieved only by affirming a suitable version of CTA” (Bishop 1989, p. 59).

15 Similarly, accepting CTA will make it very difficult to consistently defend the luck objection in order to establish that *determinism* is positively required for free will.

16 Indeed, there is no lack of compatibilist accounts of what it means to ‘take a stand’ in this way. Many compatibilists argue that such self-determination or authenticity, rather than indeterminism, is what we should really care about. See, e.g., Frankfurt (1971).

17 That this is also the case for the other major family of libertarian accounts—the so-called agent-causal theories—we will argue in Sect. 6.

18 We do not mean to suggest that there cannot be mental intentional actions that do not involve bodily movement. If one wants to affirm that possibility, ‘bodily movement’ here should be read in a broad sense, as possibly including, e.g., neural activity.

19 This diagnosis of the libertarian predicament nicely aligns with the observation in Sect. 3 that libertarians who accept CTA in order to solve the luck problem can no longer consistently accept the consequence argument. It is no accident that Bishop, who as we have seen argued against the consequence argument on the basis of CTA, also endorses the idea that freedom is intrinsic to intentional action.

20 Agent causation is even said to be “more puzzling than the problem it is supposed to be a solution to” (van Inwagen 1986, p. 151).

21 Thomas Reid, who developed an early modern theory of agent causation, for example writes in a letter to Lord Kames: “I am not able to form any distinct conception of active power but such as I find in myself [...] But, if there is anything in an unthinking inanimate being that can be called active power, I know not what it is, and cannot reason about it” (Reid 1895/1983, p. 59).

22 More recently O’Connor (2014) has suggested that all causation is substance-causation, which would imply that there cannot be intentional activity that lacks substance causal features. However, if all causation is substance causation then that makes our worry that substance-causal sourcehood does not satisfactorily capture agential control all the more pressing.

23 Similarly, when O’Connor argues for agent-causalism over event-causalism he writes: “While agents, on [the event-causal libertarian] account, do not have any less control over what they do than agents in a corresponding deterministic scenario, they also do not have more” (Jacobs and O’Connor 2013, p. 179).

24 See, e.g., Markosian (2012).

25 Of course, at this point the agent-causalist might seek to argue for indeterminism on independent grounds. But as we have tried to show in Sect. 4 current arguments for the claim that indeterminism enhances control are not very promising.

26 Indeed many have wondered why settling would essentially involve indeterminism and have therefore proposed deterministic accounts of settling. See, e.g., Broadie (2013).

27 Steward instead suggests that agency is distinctive in that it consists in the exercise of a ‘two-way power’. When an agent acts, she also always has the power to refrain from acting (Steward 2012, pp. 156, 160). This suggestion by itself, however, does not help very much in the absence of a further account of what it is to refrain from acting. For without such a story, it is unclear why a radium atom would not possess the two-way power to decay or not to decay.

28 Certainly, those who are convinced that CTA is the correct theory of intentional action will not think so. However, it should be noted that CTA is increasingly facing opposition from within the philosophy of action. See, e.g., Thompson (2008) and Lavin (2013).

## References

[CR1] Bishop J (1989). Natural agency: An essay on the causal theory of action.

[CR2] Bourget D, Chalmers DJ (2014). What do philosophers believe?. Philosophical Studies.

[CR3] Broadie S (2013). Agency and determinism in a metaphysics for freedom. Inquiry.

[CR4] Buchak L (2013). Free acts and chance: Why the rollback argument fails. The Philosophical Quarterly.

[CR5] Clarke R (2003). Libertarian accounts of free will.

[CR6] Davidson D (1963). Actions, reasons, and causes. The Journal of Philosophy.

[CR7] Davidson D, Foster L, Swanson JW (1970). Mental events. Experience and theory.

[CR8] Davidson D, Honderich T (1973). Freedom to act. Essays on freedom and action.

[CR9] Fischer JM, Kane R, Pereboom D, Vargas M (2007). Four views on free will.

[CR10] Frankfurt HG (1971). Freedom of the will and the concept of a person. Journal of Philosophy.

[CR11] Franklin CE (2011). Farewell to the luck (and mind) argument. Philosophical Studies.

[CR12] Franklin CE (2011). The problem of enhanced control. Australasian Journal of Philosophy.

[CR13] Jacobs JD, O’Connor T, Gibb SC, Lowe EJ, Ingthorsson RD (2013). Agent causation in a neo-Aristotelian metaphysics. Mental causation and ontology.

[CR14] Lavin, D. (2013). Must there be basic action? *No*$${\hat{u}}$$*s*, *47*(2), 273–301.

[CR15] Lowe E (2008). Personal agency: The metaphysics of mind and action.

[CR16] Markosian N (2012). Agent causation as the solution to all the compatibilist’s problems. Philosophical Studies.

[CR17] Mele AR (2006). Free will and luck.

[CR18] Nagel T (1986). The view from nowhere.

[CR19] O’Connor T (2000). Persons and causes: The metaphysics of free will.

[CR20] O’Connor T, Handfield T (2009). Agent-causal power. Dispositions and causes.

[CR21] O’Connor T, Kane R (2011). Agent-causal theories of freedom. The Oxford handbook of free will.

[CR22] O’Connor T, Palmer D (2014). Free will and metaphysics. Libertarian free will: Contemporary debates.

[CR23] Reid, T. (1895/1983). *The works of Thomas Reid*. Hildesheim: Georg Olms Verlag.

[CR24] Schlosser ME (2014). The luck argument against event-causal libertarianism: It is here to stay. Philosophical Studies.

[CR25] Slote M (1982). Selective necessity and the free-will problem. The Journal of Philosophy.

[CR26] Steward H (2012). A metaphysics for freedom.

[CR27] Stout R, O’Connor T, Sandis C (2010). Deviant causal chains. A companion to the philosophy of action.

[CR28] Thompson M (2008). Life and action.

[CR29] van Inwagen P (1986). An essay on free will.

[CR30] van Inwagen P (2000). Free will remains a mystery: The eighth philosophical perspectives lecture. Philosophical Perspectives.

